# Genomic signatures for drylands adaptation at gene-rich regions in African zebu cattle

**DOI:** 10.1016/j.ygeno.2022.110423

**Published:** 2022-07

**Authors:** Abdulfatai Tijjani, Bashir Salim, Marcos Vinicius Barbosa da Silva, Hamza A. Eltahir, Taha H. Musa, Karen Marshall, Olivier Hanotte, Hassan H. Musa

**Affiliations:** a International Livestock Research Institute (ILRI), PO 5689, Addis Ababa, Ethiopia; b Centre for Tropical Livestock Genetics and Health (CTLGH), ILRI Ethiopia, PO Box 5689, Addis Ababa, Ethiopia; c Cells, Organisms and Molecular Genetics, School of Life Sciences, University of Nottingham, United Kingdom; d Faculty of Veterinary Medicine, University of Khartoum, Sudan; e Embrapa Gado de Leite, Juiz de Fora, Brazil; f Institute of Molecular Biology, University of Nyala, Sudan; g Biomedical Research Institute, Darfur College, Sudan; h International Livestock Research Institute (ILRI), PO Box 30709, Nairobi 00100, Kenya; i Centre for Tropical Livestock Genetics and Health (CTLGH), ILRI Kenya, P.O. Box 30709, Nairobi 00100, Kenya; j Faculty of Medical Laboratory Sciences, University of Khartoum, Sudan

**Keywords:** Adaptive genetic differentiation, Insulin signalling, Fat metabolism, Desert adaptation, African zebu, Sudanese zebu, Hp, Pooled heterozygosity, *F*_ST_, population differentiation, XP-EHH, Cross population extended haplotype homozygosity, SNPs, Single Nucleotide Polymorphism, InDels, Insertion/Deletions, GIR, Gir, GAS, Gash, ARY, Aryashai, BTN, Butana, KEN, Kenana, BAG, Baggara, FLN, Fulani, OGD, Ogaden, BOR, Kenya Boran, ANK, Ankole, NDA, N'Dama, MUT, Muturu, YKT, Yakutian, WES, Western Finncattle, EAS, Eastern Finncattle, AAN, Angus, HOL, Holstein, ASZ, Asian zebu, SUD, Sudanese zebu, AFZ, African zebu, AFS, African sanga, WAT, West African taurine, EUT, European taurine, LD, Linkage Disequilibrium, PCA, Principal Component Analysis

## Abstract

**Background:**

Indigenous Sudanese cattle are mainly indicine/zebu (humped) type. They thrive in the harshest dryland environments characterised by high temperatures, long seasonal dry periods, nutritional shortages, and vector disease challenges. Here, we sequenced 60 indigenous Sudanese cattle from six indigenous breeds and analysed the data using three genomic scan approaches to unravel cattle adaptation to the African dryland region.

**Results:**

We identified a set of gene-rich selective sweep regions, detected mostly on chromosomes 5, 7 and 19, shared across African and Gir zebu. These include genes involved in immune response, body size and conformation, and heat stress response. We also identified selective sweep regions unique to Sudanese zebu. Of these, a 250 kb selective sweep on chromosome 16 spans seven genes, including *PLCH2, PEX10, PRKCZ*, and *SKI*, which are involved in alternative adaptive metabolic strategies of insulin signalling, glucose homeostasis, and fat metabolism.

**Conclusions:**

Our results suggest that environmental adaptation may involve recent and ancient selection at gene-rich regions, which might be under a common regulatory genetic control, in zebu cattle.

## Highlights

• Sudanese cattle thrive in the harshest environments of the African drylands.
• *Bos indicus* shared selected genes are involved in immune response, conformation, and heat stress response.
• Sudanese zebu-specific sweep includes genes involved in alternative adaptive metabolic strategies of insulin signalling, glucose homeostasis, and fat metabolism.
• Environmental adaptation in zebu cattle may involve recent and ancient selection at gene-rich regions, which might be under a common regulatory genetic control.

## 1 Introduction

Sudan, the largest country in Africa, acts as a corridor between North and sub-Saharan Africa along the river Nile. It comprises warm arid and semi-arid grazing lands, and it is home to the second-largest African population of indigenous livestock. The livestock sector plays a critical role in the Sudanese economy and the welfare of the whole population [1]. Approximately 41% of the 101 million livestock heads are cattle, the remaining are sheep, goats, and camels.

Sudan's main indigenous cattle is the humped *Bos indicus* (zebu) type. The breed names come from human tribes, external morphological traits, specific conformation, size, and branding. The main Sudanese cattle groups include the Northern Sudan short-horned zebu (Kenana, Butana, Baggara), the Nilotic zebu of Southern Sudan (e.g. Toposa-Murle, Mangala), and the Sanga (e.g. Dinka, and Nuer). Other cattle populations include White Nile cattle, Fuga or Dar El Reeh cattle of Northern Kordofan, Nuba Mountains cattle, Gash and Aryashai of East Sudan, and the Fulani cattle found in South-West Sudan and across the West Sahelian belt [[2], [3], [4]]. According to Epstein [5], cattle were introduced to Sudan from South Asia through the Nile Valley and the Horn of Africa. Recent molecular works have supported multiple arrival and movements of zebu cattle on the African continent, they gradually replaced or admixed with the local taurine cattle population [[6], [7], [8]]. Also, there is genetic and historical information suggesting that the present East African zebu is highly related to Asian zebu due to recent restocking [7].

Sudan's geography offers diversified agroecological conditions and climate, differing in rainfall, from as little as 75 mm of annual rainfall in the northern desert to about 1500 mm in the South-West forest. There is also a wide variation in the soil types, temperatures, and vegetation. The North part of the country is predominantly desertic, comprising part of the Libyan desert to the West and the Nubian desert to the East, separated by a stretch of the Nile Valley. With virtually no rainfall in this region, the primary water sources that support human settlements and vegetation are a few oases in the Libyan desert. In contrast, the South and West regions consist mainly of sandy plains interrupted by mountains. It extends from the Nuba Mountains to the borders with the Central African Republic and Chad. The Northeastern parts of the country experience little rainfall and are classified as semi-desertic [9]. Therefore, the grazing lands in the arid and semi-arid Sudan regions are characterised by extreme temperatures, high aridity, and feed and water scarcity [10,11]. The Sudanese cattle are among the few indigenous African cattle living in extreme dryland climatic conditions. It is expected that such environmental pressures would have left selection footprints on the genomes. Undoubtedly, the unravelling of the genetic mechanisms underlying the resilience of African cattle to their production environments is of interest not only to evolutionary biologists but necessary for improved breeding programs and conservation purposes.

Despite the decreasing cost of high throughput sequencing technology, exploring the genetic diversity and the adaptive traits of the indigenous African zebu population has been limited to a few studies. The majority of these previous reports involved the use of low to high-density genotype data, eg. Gautier et al. [12], Flori et al. [13], Makina et al. [14], Bahbahani et al. [[15], [16], [17], [18]], and Ben-Jemaa et al. [19]. A few other, but more recent studies, have utilized whole-genome sequencing, including Taye et al. [20], Bahbahani et al. [16], Kim et al. [8,21], Dutta et al. [22] and Wragg et al. [23]. Among the selective sweeps frequently reported are regions on chromosome 7 comprising *CXXC5, DNAJC18, NRG2, PROB1, PSD2, SPATA24,* and *SIL1*. Also, another region on chromosome 19, including genes such as *CAMTA2, DNAJC8, ENO3, GP1BA, INCA1, KIF1C, PFDN1, SLC25A11,* and *SPAG7,* has been often reported [8,16,24]. However, the link between these genes and phenotypes in African cattle requires further investigation and validation. While the East African zebu breeds from Ethiopia have largely been the focus of many previous genomics studies on indigenous African zebu, with up to 13 breeds studied, to the best of our knowledge, only two Sudanese zebu breeds, Kenana and Butana, have been studied at the level of the full genome [8,17,18,21].

**Fig. 1:**
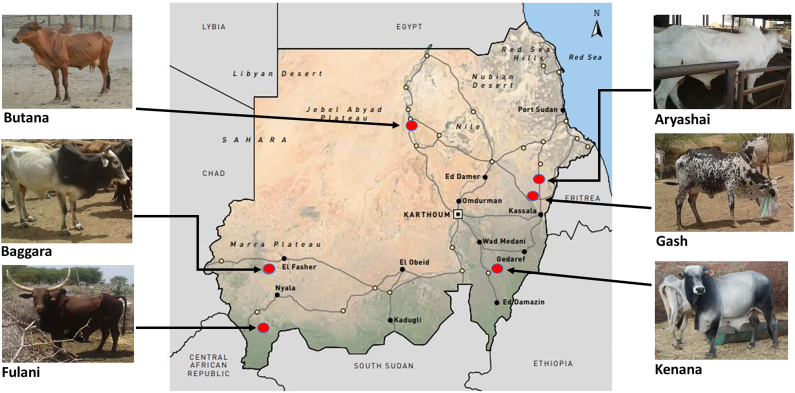
The geography of Sudan shows the sampling areas of the Sudanese zebu cattle populations. Map adapted from Impiglia [9]. Photo credit: Professor Hassan Musa, University of Khartoum, Sudan.

The present study involves whole-genome re-sequencing of six indigenous African zebu breeds sampled in Sudan (Kenana, Butana, Aryashai, Baggara, Gash, and Fulani). Kenana is found in the region between the White and Blue Nile. Butana is located mainly in the Butana plain within a triangle delimited by the River Atbara, the Blue Nile, and the River Nile. The natural habitat of Baggara cattle in Sudan is the savannah belt lying between the White Nile and the western fringes of Sudan. It is a breed associated with the Baggara Arabs ethnic group inhabiting the Sahel (mainly between Lake Chad and southern Kordofan). The Baggara nomadic pastoralists in Darfur and Kordofan moved their animals across hundreds of kilometres, searching for pastures and water. Aryashai and Gash cattle are mainly found in the El Gash delta, stretching from Aroma in the South, to Dordeib in the North and spreading to the Atbara River. Fulani cattle in Sudan are found in the western part of the country, in the Darfur and Blue Nile states (Fig. 1). The Falata Umbororo tribes mostly own Fulani cattle in Sudan [3]. The breed is also associated with the Fulani people living along the Sahelian belt. In addition, the Kenana and Butana breeds are anecdotally referred to as African dairy zebu because of their superior milk production capability. They are mostly kept under high input systems of dairy production, where the production of F1's heifer with exotic taurines for milk production is common [2].

Here, we aimed to investigate the adaptation of the Sudanese zebu population to the extreme dryland environmental conditions of the African continent, addressing the question of the genetic origin, within or from outside the African continent, of their ecological adaptations. Accordingly, we applied three genomic scan approaches to detect regions of low within-breed diversity; pooled heterozygosity (*Hp*, [25]), population differentiation (*F*_ST_, [26]), and increased haplotype homozygosity through the cross-population extended haplotype homozygosity test (XP-EHH, [27]). We performed these assessments in comparison to other cattle breeds, including East African zebu breeds (Kenyan Boran, Ethiopian Ogaden), African sanga breed (Ankole), and Gir zebu from Brazil. Other reference cattle breeds included, especially in the population structure analyses are West African taurine (N'Dama, Muturu), Eurasian taurine (Eastern Finncattle, Western Finncattle, and Yakutian cattle) and West Europe taurine (Holstein and Angus).

## 2 Results

### 2.1 Sequence reads and variants statistics

After removing adapter sequences and low-quality reads, we mapped individual sequences to the *Bos taurus* genome of reference, ARS-UCD1.2 [28]. We achieved an average alignment rate of up to 99% in all samples. Approximately 43 million SNPs were detected in all 150 cattle samples. However, the number of segregating sites (homozygous and heterozygous alternate SNPs) in each breed ranges from >23 million in the zebu (humped) breeds to between 9 and 14 million in the taurine (humpless) breeds. Within individual humped cattle, the average number of SNPs ranged from approximately nine million in Kenya Boran to 12 million in Kenana and Butana. On the other hand, a range of 4.5 million to 6.3 million SNPs was detected in the humpless African and European cattle. Therefore, the number of SNPs identified in individual humped cattle is approximately 2–2.5 times higher than the number of SNPs in individual taurine cattle (Table S2).

The estimated ratio of heterozygous to homozygous SNPs in most 17 cattle breeds is >1 as expected. The exception being the Gir (Het/Hom = 0.74), the Muturu (Het/Hom = 0.63) and the Eastern Finncattle (Het/Hom = 0.86). The observed low ratios in the Gir and finncattle could be a result of intense selection and small population sizes. In the Muturu, probably as a result of the bottleneck effect following adaptation to the tsetse-infested areas in West Africa or due to inbreeding of the endangered breed. The highest ratio among the Sudanese zebu breeds is in Butana (Het/Hom = 1.53), while the lowest is observed in Gash (Het/Hom = 1.01). The average transition versus transversion (Ts/Tv) ratio for the 17 cattle breeds is above 2.3 (Table S2).

The numbers of small insertions and deletions (InDels) in individual humped cattle range from about 1.2 million in most African and Gir breeds to approximately 1.5 million in Kenana and Butana. In comparison, the number of InDels in the taurine individuals varies between 600,000 and 800,000 (Table S2). In contrast to the database of known cattle variants (dbSNP *ver*150, last accessed December 2020), an average of 1.7 M (~ 6.5%) novel SNPs and 1.7 M (~5 0.5%) novel InDels were detected for the Sudanese population (Table S2).

### 2.2 Genetic diversity and population differentiation

**Fig. 2:**
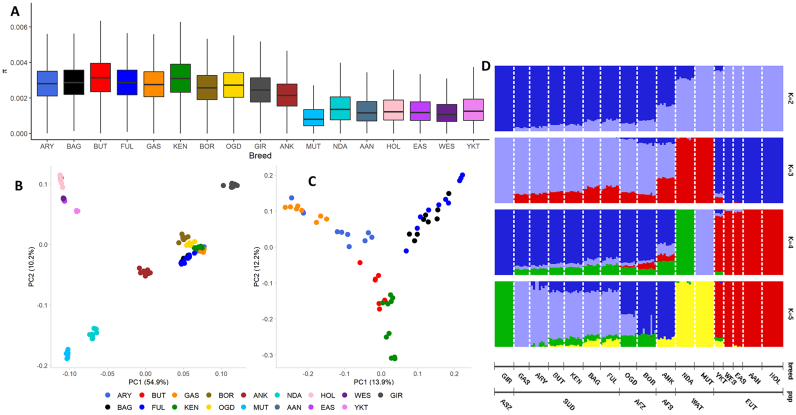
Genetic diversity and population structure of the 17 studied breeds. (A) Boxplot of average nucleotide diversities (π). The nucleotide diversity level within each cattle breed was calculated based on an overlapping 100 kb window with a 50 kb step size. (B) Principal component (PC 1 versus PC 2) analysis for 17 cattle breeds. (C) Principal component (PC 1 versus PC 2) analysis for the six Sudanese zebu cattle populations. (D) Admixture plot showing ancestry proportions for the 17 cattle breeds. The population structure was assessed using ADMIXTURE *ver*.1.3.0. The individual population is represented by a vertical bar and partitioned into coloured segments. Each segment's length represents the proportion of the inferred number of ancestries (K = 2 to K = 5). GIR – Gir, GAS – Gash, ARY – Aryashai, BTN – Butana, KEN – Kenana, BAG – Baggara, FLN – Fulani, OGD – Ogaden, BOR – Kenya Boran, ANK – Ankole, NDA – N'Dama, MUT – Muturu, YKT – Yakutian, WES – Western Finncattle, EAS – Eastern Finncattle, AAN – Angus, and HOL – Holstein. The cattle breeds were also grouped into six entities, namely, Asian zebu (ASZ), Sudanese zebu (SUD), East African zebu (AFZ), African sanga (AFS), West African taurine (WAT), and European taurine (EUT).

The average nucleotide diversities among 17 cattle breeds studied are the highest in the eight African zebu breeds, followed by the Gir, the African sanga, Ankole, and the taurine. The lowest value is observed in Muturu (Fig. 2A). Among the African zebu breeds, nucleotide diversities are the highest in the Sudanese Butana and Kenana (Fig. 2A).

Population differentiation (*F*_ST_) is generally low among the Sudanese zebu breeds (average *F*_ST_ = 0.015) and between the latter and East African zebu breeds (average *F*_ST_ < 0.041). Population differentiation between each of the African zebu and the Gir cattle is around 0.1 (Table S3A). Between the Sudanese zebu and the taurine breeds, the average population differentiation is highest between the Sudanese Gash and the taurine breeds (average *F*_ST_ = 0.324). It is the lowest with the Sudanese Baggara (average *F*_ST_ = 0.267) (Table S3A). Interestingly, the average *F*_ST_ between the African zebu and the West African taurine is higher for the West African shorthorn, Muturu (*F*_ST =_ 0.342), than the West African longhorn N'Dama (*F*_ST =_ 0.245) (Table S3A). Although we estimated the genetic parameters using a random subset of five samples in each breed, we did not observe any difference in the estimated values when we used the all available samples in each breed (see Table S3B for additional *F*_ST_ results).

### 2.3 Population structure

The principal component analysis of the 17 cattle breeds was performed using approximately 24 million autosomal bi-allelic SNPs after removing SNPs with a minor allele frequency of <0.05. The first and second principal components account for 54.9% and 12.2% variations. PC 1 clearly distinguished the taurine from the zebu breeds, while PC 2 separates African breeds from non-African breeds (Fig. 2B). A second PCA, including only the Sudanese zebu, reveals pairs of clusters (Kenana - Butana, Aryashai - Gash, and Baggara – Fulani), with no clear differentiation between the Baggara and the Fulani. PC 2 (12.1%) shows the separation of Kenana and Butana (African zebu dairy breeds) from the other Sudanese zebu (Fig. 2C).

After LD pruning (r^2^ > 0.5), a dataset of approximately 4.1 million SNPs was used to explore the proportions of genomic subpopulations (K) of the different cattle breeds for K ranging from 2 to 5 (Fig. 2D). The admixture cross-validation (CV) option [29] supports K = 4 with the lowest CV error as the likely optimum number of genomic subpopulations among the studies breeds. The four possible subpopulations inferred here are *Bos indicus*, European *Bos taurus,* and two distinct African *Bos taurus* ancestries (N'Dama and Muturu). At this level, the African zebu breeds show admixed zebu and taurine genetic backgrounds, as in previous reports [8], including two distinct African *Bos taurus* shared ancestries, the longhorn N'Dama and the shorthorn Muturu [30]. All the African zebu samples show a higher shared West African longhorn genetic background than West African shorthorn one. In addition, we observed varying low proportions (< 0.15) of shared European taurine background in all the East African zebu samples and a few Sudanese Fulani and Baggara animals (*n* ~ 3) with very low proportions of European taurine background (< 0.02) (Fig. 2D, Figs. S1 and S2). At K = 5, we observe two separate African zebu subpopulations, whereby the dryland Sudanese zebu are distinct from the East African zebu breeds (Fig. 2D). Similar to PCA results, at K = 17, we observe clustering of Sudanese zebu breeds in pairs (Fig. S3).

Both PC analyses (Fig. 2C) and admixture results (Figs. 2D and S3) indicate substantial genetic similarity between the Sudanese Baggara and the trans-Sahelian Fulani, with no separation between the two populations in the PC analysis and very similar ancestral background (admixture analysis).

### 2.4 Evolutionary relationships among cattle breeds

**Fig. 3:**
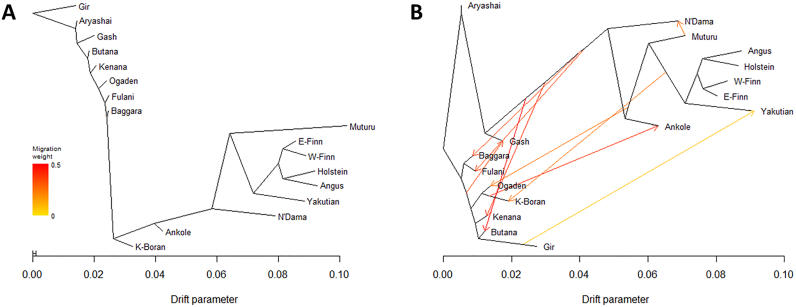
Maximum likelihood evolutionary tree and possible gene flow among the 17 cattle breeds: (A) without migration events, (B) assuming ten migration events. *E*-Finn (Eastern Finncattle) W-Finn (Western Finncattle), K-Boran (Kenyan Boran).

As for the admixture analyses, genome-wide unlinked autosomal SNPs were used for the Treemix analyses. The phylogenetic relationships and migration events reveal several possible gene flows among the cattle breeds (Fig. 3). By sequentially adding up to 10 migration events and agreeing with the admixture analysis, we observe possible gene flow from the African taurine into most African zebu breeds, including four Sudanese populations (Kenana, Butana, Baggara, and Fulani). On the other hand, we did not observe gene flow between the Sudanese zebu and any European breeds. Our Treemix results also reveal possible introgression between the two West African taurine breeds with the direction of gene flow from the shorthorn Muturu to the longhorn N'Dama (Fig. 3).

### 2.5 Genomic signatures of positive selection in zebu populations

**Fig. 4:**
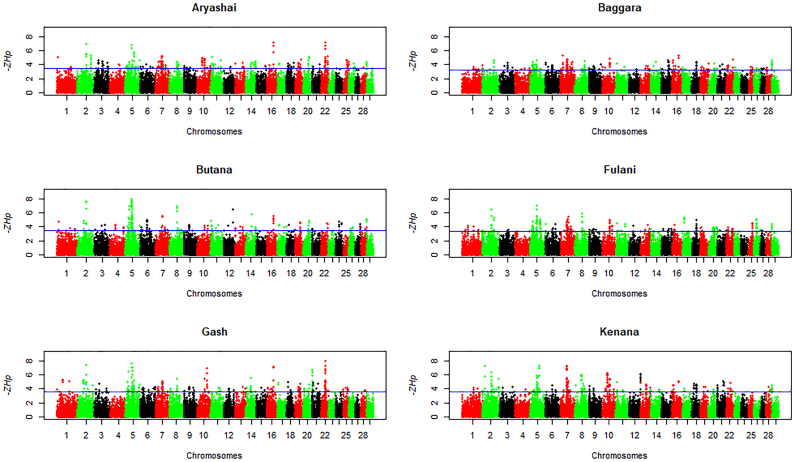
Genome-wide distribution of *ZHp* scores across bovine autosomes in six Sudanese zebu populations. The blue line indicates the *ZHp* threshold value (lowest 0.5%) for selecting outlier windows (candidate regions under positive selection). (For interpretation of the references to colour in this figure legend, the reader is referred to the web version of this article.)

We performed a genome-wide autosomal *Hp* selection scan in the nine zebu breeds (six Sudanese zebu populations, Kenyan Boran, Ogaden, and Gir). The candidate regions for positive selection will have extreme negative *Z*-transformed *Hp* scores, as shown in Fig. 4 (Sudanese zebu breeds) and Fig. S4 (other zebu breeds). We considered 249 outlier windows (100-kb windows in the lowest 0.5% of *ZHp* scores) in each breed as regions with significantly reduced diversity and possible signatures of positive selection (Tables S4–S12). We identify 273 outlier windows common to at least two Sudanese zebu breeds. We merged the windows in the proximity of up to 5 kb, resulting in 114 selective sweeps regions varying in size from 0.1 to 1.5 Mb. Fifty-five selective sweep regions overlap with outlier windows detected in at least one non-Sudanese zebu population. The remaining 59 selective sweep regions were detected exclusively in the Sudanese zebu populations. Thus, these regions are shared and unique Sudanese zebu selective sweep regions, respectively (Table S13). Based on *Ensembl* cow genes 104 (ARS-UCD1.2), 47 out of the 55 shared Sudanese – non-Sudanese zebu selective sweep regions overlap with 227 protein-coding genes (Tables S14). In contrast, of the 59 unique Sudanese selective sweeps, 40 overlap with 135 protein-coding genes (Tables S15). The selective sweep regions in the Sudanese zebu populations devoid of any annotated cow genes span a total 4.15 Mb ARS-UCD1.2 genome region (Table S16). The functional annotation of the individual Sudanese zebu and or combined gene sets reveal a few statistically significance (≤ 0.05, Benferoni correction) enriched biological processes in five of the Sudanese zebu breed except Fulani (Table S17).

### 2.6 Shared African and Gir zebu selective sweep regions

Among the 47 shared selective sweep regions overlapping annotated genes, two regions, on chromosome 5 (5:47.40–48.0 Mb) and 7 (7:49.65–51.15 Mb), are detected in eight of the nine zebu breeds. We detect the 600 kb region on chromosome 5 in all the six Sudanese zebu populations, the Kenyan Boran and Gir breeds but not in the Ethiopian Ogaden. The region overlaps eight genes (Table S14), with functions related to growth, conformation, reproduction, and the immune systems, some of which are previously reported in cattle [24,31]. Likewise, the ~1.5 Mb gene density sweep on chromosome 7 overlaps with up to 28 protein-coding genes, including two previously reported heat shock proteins, *DNAJC18* and *HSPA9* (Table S14)*.* Again, this large sweep is detected in eight zebu breeds, except the Sudanese Butana. Other highly common, shared selective sweep regions between African and non-African zebu breeds (present in at least six breeds) are found on chromosomes 2 (2:70.25–70.50 Mb), 8 (8:59.35–59.70 Mb), 10 (10:58.85–59.15 Mb), and 17 (17:13.15–13.35 Mb) (Table S14).

We found several candidates selected regions in African zebu breeds which are not present in the Gir. These include regions on chromosomes 7 (7:52.10–52.40 Mb), 11 (11:13.10–13.30 Mb), and 20 (20:48.80–48.95 Mb) detected in seven of the eight African breeds. The exceptions are Butana (chromosome 7), Gash (chromosome 11), and Kenana (chromosome 20). In particular, the region on chromosome 7 overlaps with up to eight protein-coding genes (Table S14). Also, we found another gene density region on chromosome 19 (19:26.35–26.50 Mb), containing up to fourteen protein-coding genes detected in six African zebu breeds, including four Sudanese populations (Table S14).

Besides, we identified regions in the Sudanese zebu population shared only with the Gir but not with other African zebus. These include a 650 kb region on chromosome 5 (5:48.4–49.05 Mb) found in five Sudanese zebu populations, the exception being the Baggara. This region overlaps five genes (*ENSBTAG00000000237, LEMD3, MSRB3, TBC1D30,* and *WIF1*)*.* Also, a 150 kb candidate selected region on chromosome 2 is detected in four Sudanese populations and the Gir. It overlaps with the macrophage receptor with the collagenous structure gene (*MARCO*), involved in the innate immune response [32]. Additionally, a 150 kb region on chromosome 5, overlapping with the *HOXC* gene cluster, was also detected exclusively in four Sudanese zebu populations and the Gir (Table S14).

### 2.7 Sudanese zebu specific candidate selective sweeps

We classified as Sudanese zebu-specific sweeps, 59 genome regions of low diversity (*ZHp*) detected uniquely in the Sudanese zebu populations. The commonest sweep regions are a 450 kb region on chromosome 7 (7:54.0–54.45 Mb) and a 250 kb region on chromosome 16 (16: 50.40–50.65 Mb). They are present in five Sudanese breeds, the exception being Baggara and Fulani, respectively (Table S15). The region on chromosome 7 contains *ARHGAP26* and *NR3C1*, while chromosome 16 region contains seven genes (*FAAP20, MORN1, PEX10, PLCH2, PRKCZ, RER1,* and *SKI*). In addition, we identified in four Sudanese breeds a 200 kb region on chromosome 7 (7:19.85–20.05 Mb). This region is downstream of another 150 kb region (7:19.60–19.75 Mb) detected in three Sudanese populations. The two regions are close to each other, being only separated by 100 kb. Together, they overlap with 16 genes (Table S15). More so, a high density gene region of 200 kb on chromosome 5 (5: 55.85–56.05 Mb) was detected in three populations. It includes seventeen genes (*ARHGAP9, ARHGEF25, B4GALNT1, DCTN2, DDIT3, DTX3, ENSBTAG00000049386, ENSBTAG00000051574, ENSBTAG00000051593, GLI1, INHBC, INHBE, KIF5A, MARS1, MBD6, PIP4K2C,* and *SLC26A10*). The remaining regions overlapping at least one protein-coding gene are detected only in atmost two Sudanese zebu breeds (Table S15).

### 2.8 Genetic differentiation at shared and unique Sudanese zebu candidate selected genome regions

**Fig. 5:**
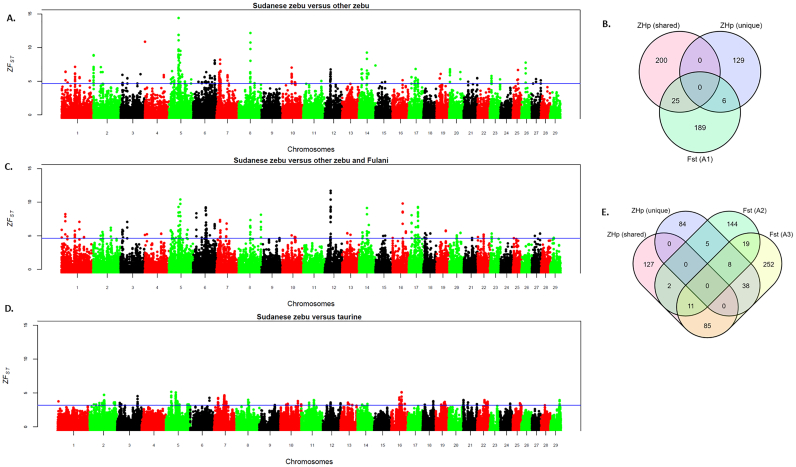
Genome-wide distribution of *ZF*_ST_ scores along the *Bos taurus* (ARS-UCD1.2) autosomes and overlap of detected protein coding genes following the population differentiation *F*_ST_ analyses between Sudanese zebu populations and the populations of other cattle breeds. (A) *F*_ST_ analysis between population of six Sudanese zebu breeds and other zebu/sanga (Kenya Boran, Ogaden, Ankole and Gir). (B) Venn diagram showing the overlap of detected protein-coding genes between *ZH*p and *F*_ST_ tests in (A). (C) *F*_ST_ analysis between five Sudanese zebu breeds (except Fulani) and the population of other zebu/sanga comprising Fulani, Kenya Boran, Ogaden, Ankole and Gir). (D) *F*_ST_ analysis between population of five Sudanese zebu breeds (except Fulani) and the population of all taurine breeds studied. The blue lines indicate the top 0.5% *ZF*_ST_ values threshold to consider outlier regions. (E) Venn diagram showing the numbers of overlapping detected protein-coding genes between *ZH*p and *F*_ST_ tests in (C) and (D). (For interpretation of the references to colour in this figure legend, the reader is referred to the web version of this article.)

Further, we searched for unique selective sweeps in the Sudanese population possibly linked to environmental adaptation in the arid and semi-arid African regions. Hence, we looked for evidence of differentiation in genome regions between the populations of the Sudanese zebu, and the other cattle populations studied, following the Weir and Cockerham [33] genetic differentiation index (*F*_*ST*_) test. The genome-wide distribution of *Z*-transformed *F*_*ST*_ values across the 29 bovine autosomes for the initial *F*_ST_ (A1) is presented in Fig. 5A. We identified 236 outliers 100-kb windows in the top 0.5% *ZF*_*ST*_ values (0.1 ≤ *F*_ST_ ≤ 0.3 and 4.6 ≤ *ZF*_ST_ ≤ 14.3). Neighbouring windows were merged, resulting in 62 selective sweeps spanning 220 protein-coding genes (Table S18).

By comparing *F*_ST_ and *ZHp* detected candidate gene sets, we found an overlap of 31 protein-coding genes, which we had initially classified as Sudanese zebu shared (*n* = 25) and unique (*n* = 6) candidate positively selected genes (PSGs) (Fig. 5B, and Table S19). Of the 25 highly differentiated, shared PSGs, only one is found on chromosome 8, the remaining 24 are found within five selected sweeps on chromosome 5. Three of these regions are nearby within (5:47.05–49.25 Mb), and add up to a 1.75 Mb length. However, the six unique candidate PSGs are found on chromosomes 7 (*n* = 3) and 16 (*n* = 3). These genes include *STAP2, ARHGAP26, NR3C1, FAAP20, MORN1* and *SKI.* (Table S19). Interestingly, the latter five genes are under selection in up to five Sudanese breeds, the exception being Baggara for the chromosome 7 genes (*ARHGAP26* and *NR3C1*) and the Fulani for the chromosome 16 genes (*FAAP20, MORN1* and *SKI*) (Table S15). While the chromosome 16 candidate selected genes are found within a 100 kb selective sweep as detected by *F*_ST_, the region extends up to 250 kb based on *ZH*p. It overlaps seven protein coding genes (Table S15). We hypothesize that these genes being present in sleective sweep regions detected exclusively in a majority of Sudanese zebu population could be linked to their unique phenotype, possibly to specific adaptation in the arid and semi-arid regions.

Further, we performed two additional *F*_ST_ analyses with the aim to further classify the Sudanese zebu selective sweeps as candidate selected region in comparison to other zebu breeds, taurine cattle or both. We identified 225 and 249 outliers 100-kb windows in the top 0.5% *ZF*_ST_ values for the zebu and taurine comparison analyses. Neighbouring windows were merged, resulting in 71 and 87 selective sweeps, spanning 189 and 413, respectively protein-coding genes, respectively (Tables S20 and S21, Fig. 5B–C). Next, based on the overlaps of *ZH*p results and at least one of the two latter *F*_ST_ results (Fig. 5E), we classify detected PSGs in the present study as *Bos indicus* specific, African zebu specific and Sudanese zebu specific (Table S22). The majority of the *Bos indicus* specific PSGs (*n* = 85) span selective sweep regions of high *F*_ST_ between zebu and taurine located on chromosoms 5, 7 and 22. African zebu specific selective sweep are located on chromosome 13 and 19 (Table S22).

On the other hand, Sudanese-specific PSGs spans selective sweeps and regions of high *F*_ST_ between Sudanese zebu population and other studied cattle populations (zebu or taurine specific or both) and are mostly located con chromosomes 5, 7,10 and 16. (Table S22). While some of these regions are gene-rich (containing minimum of five protein coding genes) and could contain important adaptive or production genes targeted by selection, they are less frequently (< 4) detected in the sudanese population. However, the 250 kb selective sweep locus on chromosome 16 is the most frequent as it is detected in five of the Sudanese zebu breeds and is gene-rich, spanning seven genes; *FAAP20, MORN1, PEX10, PLCH2, PRKCZ, RER1*, and *SKI* (Table S22). Indeed, we hypothesize that these highly frequent chromosome 16 sweep could be linked to the adaptation of cattle to the African dryland region of Sudan.

### 2.9 A unique Sudanese zebu-specific candidate selective sweep at chromosome 16

**Fig. 6:**
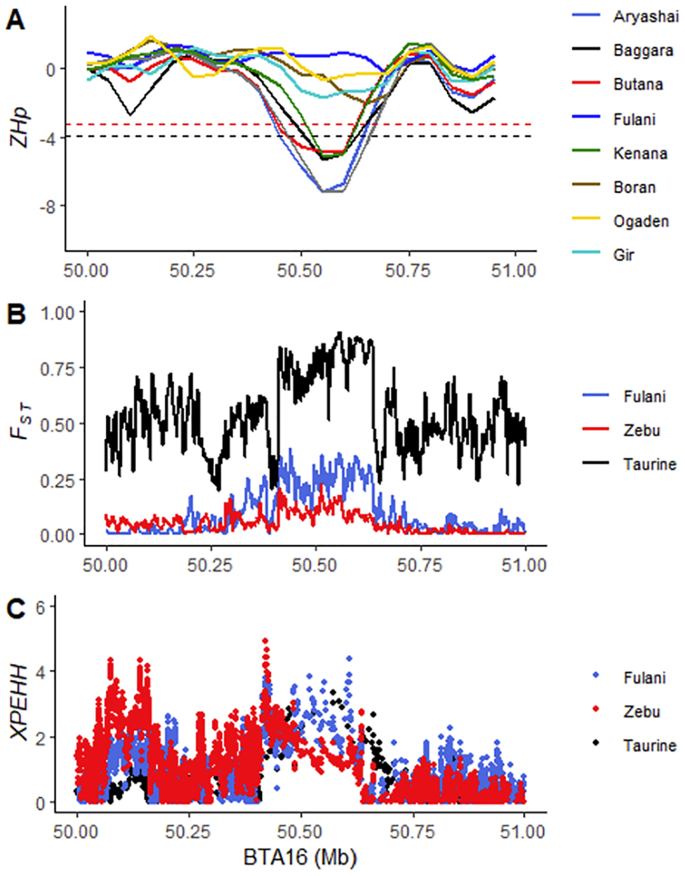
Strong evidence of positive selection in Sudanese zebu population at a 250-kb chromosome 16 locus. (A) Genomic footprints of low diversity (*ZHp*) in the candidate region in eight African zebu breeds and Gir zebu. The red and black dashed line indicates the maximum and minimum genome-wide *ZHp* selection outlier threshold values among the cattle breeds. (B) Population differentiation (*F*_ST_) between the Sudanese zebu populations (excluding the Fulani) and other zebu and taurine cattle populations. (C) Increased haplotype homozygosity (XP-EHH) at the 250 kb selective sweep locus in Sudanese zebu compared to other cattle populations. (For interpretation of the references to colour in this figure legend, the reader is referred to the web version of this article.)

**Fig. 7:**
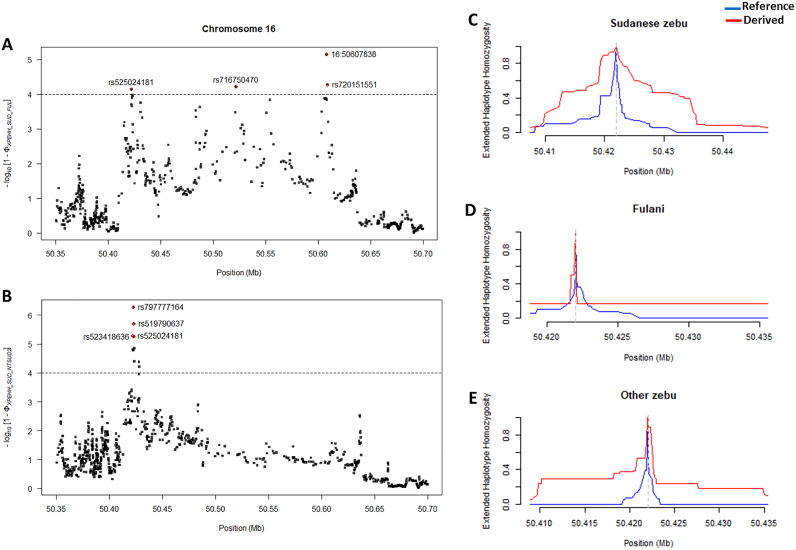
Localisation of SNPs at the Sudanese zebu-specific candidate selective sweep at the chromosome 16 locus following XP-EHH analysis between the combined five Sudanese breeds and (A), Fulani, and (B), other zebu breeds (East African and Gir breeds). (C) The decay of extended haplotype homozygosity around rs525024181 in Sudanese Zebu, (D) Fulani, and (E) other zebu breeds.

To further confirm the evidence of recent selection at the chromosome 16 locus in the Sudanese population, and possibly avoid the problem of confounding demographic factors [34], we performed additional genomic scan analysis on the entire bovine chromosome 16 using the XP-EHH test [27]. We used the Sudanese Fulani, the other zebu breeds, and the taurine population as three separate control groups (Fig. S5A—C). Based on the three selection scans approaches applied in this study, the evidence of selection in the region of the 250 kb selective sweep chromosome 16 locus in the Sudanese zebu population is shown in Fig. 6. The evidence of increased haplotype homozygosity in this region in the Sudanese zebu population compared to other zebu and taurine cattle populations is shown in Fig. 6C. In particular, the comparison with Fulani reveals selection signals at four significant SNPs (−log10 *XP-EHH*, > 4), which are located within three genes (*PRKCZ* (16:50607838 and rs720151551), *SKI* (rs716750470) and *PLCH2* (rs525024181) (Fig. 7A). In contrast, XP-EHH comparison with other zebu populations (East African breeds and Gir) reveals one strong signal containing several significant SNPs (Fig. 7B). The latter's top four significant SNPs (rs797777164, rs519790637, rs525024181, and rs523418636) are located within the *PLCH2* gene.

Consequently, *PRKCZ, SKI,* and *PLCH2* genes might be zebu-specific candidate genes linked to local adaptation to the dryland habitat in Sudan's desert and semi-desertic regions. Indeed, they are involved in alternative metabolic processes of glucose homeostasis, insulin signalling, and fat metabolism, which are likely relevant adaptive strategies in hot and arid environments [[35], [36], [37], [38], [39]]. Furthermore, we considered the one common significant SNP (rs525024181, G > A) within the *PLCH2* genes (Fig. 7A–B) as a putative candidate variant or closely linked polymorphism targeted by selection. The haplotype decay around rs525024181 reveals that the haplotype containing the derived allele has more extensive homozygosity in the Sudanese zebu than in the other zebu breeds (Fig. 7C–E), suggesting a strong selection at the locus in the Sudanese population. We did not observe much difference in the pattern of EHH around the remaining seven significant SNPs among the zebu populations (Fig. S6).

## 3 Discussions

In this study, we analyse for the first time the whole-genome sequences of six indigenous African zebu breeds from the most extreme African dryland conditions of Sudan. We aimed to investigate population genetic structure and identify candidate selective sweep regions linked to dryland adaptive traits and to assess their uniqueness among other African and non-African cattle. Our within Sudanese population PCA and admixture at K = 17 results show clusters of the Sudanese breeds in pairs. The Butana-Kenana are two breeds with superior milk production potential among the Sudanese zebu population. The Baggara-Fulani relatedness may be the result of common ancestry following movement westward of zebu along the Sahelian belt. They both show higher taurine genomic component including European taurine background, compared to others Sudanese zebu. Intriguingly, the separation of the Sudanese zebu from the East African zebu based on the our admixture result supports the two waves of zebu entry to Africa continent. However, these results would need to be further investigated with large sample size and wider geographical coverage of the continent.

We adopted three genomic scan approaches, detecting genomic regions of reduced diversity (*ZHp*), population differentiation (*F*_ST_), and increased haplotype homozygosity (XP-EHH). Most of the within-breed *ZHp* genomic signatures are unique to individual zebu breeds. Interestingly, we find several gene-rich regions among the shared candidate selective sweeps across Sudanese or between Sudanese and other zebu breeds. Also, some of these regions show high population differentiation signals among the cattle breeds studied. However, a few of the identified selective sweeps are being rather African dryland specifics as detected exclusively in the Sudanese cattle population of the arid environments. We define gene-rich region in our study as a selective sweep region with more than five genes whether or not belong to the same functional pathway. If under common regulatory control, the identified gene-rich regions will support the view that change in gene expression in a gene-rich region may control the expression of multiple phenotypes [40] providing a possible mechanism for rapid adaptation to new environmental challenges [41].

Interestingly, the functions of the genes within candidate gene-rich regions identified in the present study are associated with phenotypes such as body size and conformation, stress response to heat, immune response, insulin signalling, glucose metabolism, and fat metabolism. Positively selected genes involved in similar biological processes have been reported in other desert-dwelling mammals like sheep, goats, and camels, but many these reported genes are not the same [[42], [43], [44]]. However, we identified a few overlap between the present study and previous studies involving Sudanese cattle and camel as reported by Bahbahani et al. [17,18,45]. (Table S22) evidence for common adaptive traits or mechanisms among the ungulates adapted to hot and arid climates [39,46,47].

Among the *Bos indicus* specific selective sweeps commonly detected across zebu populations, we found candidate genes involved in body size, conformation, stress response to heat, and the immune response. Notably, the pleiotropic *HMGA2* gene within the expanded selective sweep on chromosome 5 has been linked to different phenotypes, including growth and conformation in other non-African cattle breeds [24,31,[48], [49], [50]], and other mammalian species ([[51], [52], [53], [54]] [22,55]). Exposure to infectious and parasitic disease challenges is another stressor faced by Sudanese cattle living in hot arid environments, necessitating an adequate immune response for survival in the region. We found the interleukin 1 receptor-associated kinase 3 (*IRAK3*) gene in the proximity of the *HMGA2* gene. *IRAK3* is a crucial modulator of inflammatory responses and negatively regulates Toll-like receptor signalling pathways involved in innate host defence [56]. It has been reported under positive selection in non-African humped cattle adapted to tropical climatic conditions [24,31].

On the contrary, several genes overlapping the extended gene-rich selective sweep on chromosome 7 have previously been detected in both African and non-African zebu breeds ([8,16,21,24]. Kim et al. [8] reported an excess of indicine ancestry in this later region. Furthermore, among the twenty-eight genes in this region is the DnaJ heat shock protein family (HSP40) member C18 (*DNAJC18*), also reported in tropically adapted sheep breeds from Ethiopia [57] and Bactrian camel [42]. Also, selective sweep spanning the region of the Homeobox cluster locus has been reported in Gir cattle [58], Chinese zebu [59] and recently the paralog *HOXD* cluster was reported in West African zebu [60]. This regions may be of importance for the subspecies, no matter the particular history of different local zebu populations, and could contribute to partially explaining the well-known ability of zebu cattle for resistance to tick attacks. Overall, the *Bos indicus* specific selective sweep genes could be involved in zebu resilience to the tropics. However, these region requires further investigation and fine mapping to identify the probable candidate genes or variants targeted by selection.

In addition to extreme temperatures, other harsh conditions of the African dryland areas include food and water shortages [61,62]. Indeed, we identified a unique Sudanese zebu selective sweep on chromosome 16. This region also shows high population differentiation between the Sudanese zebu and other cattle populations, including the Sudanese Fulani. The absence of this selection signal in the Fulani will need to be further investigated, considering the genetic closeness of the Fulani and Baggara and the origin of the Fulani breed, predominantly found in the West African Sahelian region [2,3]. The candidate genes overlapping this unique sweep have roles in glucose homeostasis, feed efficiency, insulin signalling, and fat metabolism. Glucose homeostasis and insulin signalling are essentially biological processes contributing to animals' survival due to nutritional shortages in the desert. Also, there are pieces of evidence of the periods of dietary restriction in cattle and other species coinciding with increased insulin sensitivity and glucose uptake [[63], [64], [65], [66]]. In addition, *FAAP20, PRKCZ,* and *SKI* genes have been implicated in insulin metabolism [35,36,[67], [68], [69]]. In particular, *PRKCZ* functions by regulating the translocation of the glucose transporter 4 (*GLUT4*) to the cell surface for glucose uptake and has been implicated in insulin response to glucose uptake in fasted cattle. Thus, a greater expression of both *PRKCZ* and *GLUT4* has been observed in fasted animals, suggesting a greater sensitivity to glucose uptake by myocytes [70].

Our results suggest shared physiological responses facilitated by glucose transporter proteins in camelids and cattle during water and food scarcity. Indeed, increased expression levels of *GLUT1* (glucose transporter 1) and genes involved in glycolysis in the renal medulla have been reported in water-deprived Bactrian camels [42]. However, the processes may have evolved more rapidly in camelids than in cattle as a result of *GLUT1* being ubiquitously expressed than *GLUT4*, which is expressed mainly in insulin-sensitive tissues like the heart, skeletal muscle, and adipose tissue [42,71]. In addition, increased expression of the insulin-responsive glucose transport proteins has been correlated with improved insulin action in skeletal muscle, especially during exercise. Overexpression of the *SKI* gene in the skeletal muscle has been demonstrated to modulate the genetic controls of insulin signalling and, thus, glucose homeostasis [35,72].

The above function may correlate with whole-body fat reduction in exercised humans due to increased insulin sensitivity of triglyceride lipolysis in subcutaneous adipose [72]. Also, data from transgenic animals have further supported the importance of adipose tissue in modulating whole-body insulin sensitivity [73]. Hence, the role of adipose tissue mediated by lipid metabolism in the physiological adaptation process to hot arid environments has been well documented. Furthermore, adipose tissue serves as an organ for storing food and a source of energy and water, contributing to animal survival during prolonged starvation and thirst. Moreso, fat oxidation produces metabolic water in water-deprived or exercised animals [74]. Strikingly, two other genes, *PLCH2* and *PEX10,* found within the unique Sudanese zebu selective sweep on chromosome 16, are involved in the adaptive metabolic strategy of lipid catabolism and oxidation. Functionally, the phospholipase C eta 2 (*PLCH2*) gene, also known as *PLCL4,* among other aliases, is suggested to participate in the lipid catabolic process for glucogenesis. Moreover, the hypermethylation of loci associated with *PLCH2* coinciding with fat reduction has also been reported in calory restricted humans [75]. On the other hand, peroxisomes such as *PEX10* control the composition of intracellular fatty acid content especially, the unsaturated fatty acid content, and are essential in the oxidation of fatty acids to produce water [[76], [77], [78]].

## 4 Conclusions

The present study analysed the whole genome re-sequencing of indigenous Sudanese zebu breeds as a proxy to understanding cattle adaptations to environmental challenges in the African dryland area. Our results align with previous reports on other mammalian species enabling adaptation to similar conditions of the harsh desert environment. Also, we highlight the importance of selection at gene-rich genome regions as a possible mechanism of rapid adaptation to the complexity of environmental challenges, more importantly, if these gene-rich regions are under the genetic control of a similar regulatory mechanism. This will require further investigation.

## 5 Materials and methods

### 5.1 Study population and sample re-sequencing

A total of 17 zebu and taurine cattle breeds comprising 155 individuals were included in the present study (Table S1). We newly generated the full genome sequences of indigenous Sudanese zebu cattle consisting of 60 individuals of Kenana (KEN, *n* = 10), Butana (BTN, *n* = 10), Aryashai (ARY, *n* = 10), Baggara (BAG, *n* = 10), Gash (GAS, *n* = 10) and Fulani (FLN, *n* = 10). Also, we included new sequences of 10 Gir cattle from Brazil (GIR, *n* = 10). The remaining 85 genome sequences are from public databases. They include Ogaden (OGD *n* = 9, Ethiopia), Kenyan Boran (BOR *n* = 10, Kenya), Savannah Muturu (MUT *n* = 10, Nigeria), N'Dama (NDM *n* = 10, The Gambia), Ankole (ANK, *n* = 10, Uganda), Angus (ANG, *n* = 10), Holstein (HOL, *n* = 11), Eastern Finncattle (EFN *n* = 5) Western Finncattle (WFN *n* = 5) and Yakutian (YKT *n* = 5) [16,21,79,80] The NCBI SRA accession numbers are provided in Table S1.

The Sudanese dataset was generated as part of the project on “agricultural growth, capacity building for scientific preservation of livestock breeds in Sudan”. The ethical committee of the Faculty of Veterinary Sciences, University of Nyala, Sudan, approved the sampling protocol for the Group A Sudanese cattle. We obtained 10 ml of whole blood from each animal into EDTA VACUETTE® tubes following the standard procedure under veterinarian supervision. According to the manufacturer protocol, genomic DNA was extracted from the blood samples using the Qiagen DNeasy extraction kit (Qiagen, Valencia, CA, USA). Genomic DNA was evaluated using a NanoDrop spectrophotometer (NanoDrop Technologies, USA) and gel electrophoresis. Paired-end sequencing was performed on the Illumina® HiSeq platform, with the read length of PE150 bp at each end.

The Gir cattle samples were provided by Embrapa Dairy Cattle, Brazil. The samples were part of the progeny test program from the National Program for Improvement of Dairy Gir (PNMGL), headed by Embrapa Dairy Cattle (Juiz de Fora, Minas Gerais, Brazil) in cooperation with the Brazilian Association of Dairy Gir Breeders (ABCGIL) and the Brazilian Association of Zebu Breeders (ABCZ). Semen samples were collected for DNA extraction. For this purpose, the semen samples were washed with a lysis buffer and incubated for 2 h with an extraction buffer containing dithiothreitol 10% and RNase. Pellets were incubated overnight with a saline-proteinase K buffer, and a phenol-chloroform extraction removed the proteins. The quality and quantity of DNA for all samples were evaluated using a NanoDrop 1000 spectrophotometer (Thermo Scientific, Wilmington, DE, USA). Mate-paired and paired-end libraries (200 bp and 2 × 100 bp, respectively) with different insert sizes were prepared according to the Illumina protocol and subsequently sequenced on the Illumina® HiSeq platform (Illumina Inc., San Diego, CA, USA).

### 5.2 Reads alignment, variants discovery, and quality control

We subjected individual sample raw reads to initial quality control using Trimmomatic *v*0.38 [81]. First, we trimmed paired reads of adapter, low-quality bases (qscore <20) at the beginning and end, then filtered out reads with mean q score <20 or length <35 bp. The final quality of the resulting clean reads was confirmed using FastQC v0.11.5 (https://www.bioinformatics.babraham.ac.uk/projects/fastqc/). Next, trimmed sequences were aligned to the *Bos taurus* reference genome ARS-UCD1.2 [28], using BWA-MEM v0.7.17 [82] with default parameters. SAMtools *ver* 1.9 was used to convert SAM files to BAM format, and for sorting by contigs [83], duplicates were marked using Picard tools *ver* 2.18.2 (http://broadinstitute.github.io/picard/).

After mapping, we processed the resultant alignment files and performed variants (SNPs and insertions/deletions) discovery following the Genome Analysis Toolkit best practices pipeline (https://gatk.broadinstitute.org/hc/en-us/sections/360007226651-Best-Practices-Workflows). Here, variant calling was performed on individual cattle samples using the Haplotype caller of GATK v3.8–1-0-gf15c1c3ef [84] and incorporating known variants from dbSNP *ver*150 [85]. The GATK joint genotyping approach (*GenotypeGVCFs* mode) was adopted to identify variants in all cattle samples simultaneously. We performed two joint genotyping analyses on separate datasets. The initial one involved 155 animals, including the 60 Group A Sudanese samples and 95 non-Sudanese individuals. The identified SNPs and insertions/deletions (InDels) were separately subjected to GATK hard filter (VariantFiltration) steps. The filtering criteria for SNPs include (QD > 2.0, MQ > 40, ReadPosRankSum >8.0, HaplotypeScore >13, MappingQualityRankSum >12.5). The autosomal biallelic SNPs that passed the above filtering criteria with a Phred-scaled quality score of above 20 (QUAL >20; approximately 99% likelihood of being correct) were retained for further analyses. The filter criteria for detected InDels include (QD < 2.0 || FS > 200.0 || ReadPosRankSum < −20.0 || QUAL <20).

Further, the proportion of missing genotypes in the individual sample and the relatedness among other samples were estimated using the options “*--missing-indv*” and “*—relatedness*”, respectively, of vcftools v0.1.15 [86]. Both individuals of a pair of samples with high relatedness (> 0.8) were excluded from the dataset if they belong to different breeds. In contrast, only one of two individuals with high relatedness was excluded if they belonged to the same breed. Following these criteria, five samples, including two Butana (*n* = 2), two Gash (*n* = 2), and one Baggara (*n* = 1), were removed. No animal was removed due to excessive missing data (> 20%). Hence, 150 cattle samples were retained in further analyses.

The total numbers of variants (SNPs and InDels) and the transition to transversion (Ts/Tv) ratio for individual cattle breeds were estimated using bcftools *ver* 1.8 [83]. SnpEff v4.3t [87] was used to ascertain the genome location and effects of detected variants based on the *Ensembl* cow gene database (ARS-UCD1.2) dbSNP *ver*150. The proportion of detected variants was classified as “known” if the non-reference allele is present in the dbSNP, otherwise as “novel.”

### 5.3 Genomic diversity and inbreeding

From the detected high-quality autosomal SNPs (~ 42.9 M), we estimated the counts of segregating sites for individual cattle using PLINK v2 ([88]). The average of individuals of the same breed was reported as the per breed estimate. Within-population nucleotide diversity (π) values [89] and global averages of pairwise population differentiation (*F*_ST_) [33] among cattle breeds were estimated based on overlapping 100 kb window, and 50 kb step size along bovine autosomes using vcftools *v0.1.15*. Due to the differences in the number of samples in some of the studied breeds, we randomly selected five representative samples from each breed to estimate these latter parameters.

### 5.4 Population genetic structure

We performed Principal Component Analysis (PCA), admixture, and maximum likelihood tree (Treemix) to investigate the genetic structure among the different cattle breeds using whole-genome autosomal SNPs. The initial 42.9 M SNPs were filtered based on minor allele frequency (MAF < 0.05), resulting in a dataset of about 24 M SNPs which was then used for PCA. We used PLINK software to generate PCA eigenvectors and eigenvalues in two categories: all 17 cattle breeds and Sudanese zebu samples only. PCA plots were generated for the first two eigenvectors using the ggplot2 package in the R *ver* 3.6.3 environment.

Further, linkage disequilibrium (LD) pruning of 24 M SNPs was performed using PLINK default option “50 kb step 10 kb SNPs, r^2^ > 0.5” [88], resulting in approximately 4.1 million SNPs.The block relaxation algorithm implemented in the ADMIXTURE *ver 1.3.0* software [29] was used to identify the inferred subpopulation clusters among all the individual cattle studied, with the ‘K' value set from 2 to 5 and also K = 17 equal to the number of the breed involved in this study was investigated. Admixture analysis was preceded by removing SNPs with >10% missing data (*−-geno 0.1*) using vcftools. Finally, the admixture subpopulation ratio was visualised using the pophelper R package [90].

Using the pruned dataset, phylogenetic relationships based on the maximum likelihood tree and possible gene flow events among cattle breeds were investigated using Treemix *v*1.3 [91]. The phylogenetic tree was constructed several times, incorporating possible migratory events, from m0 (no migration event) to m10 (ten migration events). Finally, the models were visualised in R using the script provided in Treemix.

### 5.5 Signatures of selection analyses and functional annotation

We applied three genomic scan approaches to detect candidate regions under positive selection, within breed diversity (*ZHp*), population differentiation (*F*_ST_), and increased haplotype homozygosity (XP-EHH).

We applied the within-population *Hp* test to detect genomic signatures of low diversity in each of the eight African zebu breeds and the Gir breed. The *Hp* analysis involves counting reads with the most major and most minor abundantly observed alleles at every SNP position within a specified window size and sliding step size. The distribution of *Hp* values was normalised by transforming *Hp* to *Z*-scores (*ZHp*) using (*ZHp* = (*Hp* - *μHp*)/*σHp*) [25,92,93]. Using vcftools [86], we estimated regions of population differentiation (*F*_*ST*_) based on Weir and Cockerham [33] between the Sudanese zebu populations and the combined African zebu, Sanga and Gir breeds (Ogaden, Kenya Boran, Ankole and Gir). We performed three genome-wide *F*_ST_ analyses. Firstly, we calculated *F*_*ST*_ between the combined six Sudanese zebu breeds and the combined population of African zebu (Ogaden, Kenya Boran), Sanga (Ankole) and Asia zebu (Gir). Next, we performed additional *F*_ST_ analyses between the population of combined five Sudanese zebu (except Fulani) and two reference populations of zebu/sanga and taurine cattle breeds in two separate *F*_ST_ analyses (A2 and A3). The zebu/sanga reference population include the other zebu as in *F*_ST_ A1 and the Fulani. The taurine reference population comprises the west African taurine, Eurasia taurine and European taurine. The weighted *F*_*ST*_ were also *Z*-transformed in R. In each *ZH*p and *F*_ST_ test, we estimated genome-wide test statistics using 100 kb windows and sliding 50 kb step across the bovine 29 autosomes and excluded windows containing <10 SNPs. Also, the 100-kb windows in the extreme top 0.5% test values were arbitrarily defined as candidate selection outlier windows. We find an overlap of detected selective sweep regions between the two *F*_ST_ analyses using BEDTools intersect (*version* 0.2.29) [94].

*Bos taurus* (ARS-UCD1.2) genes overlapping the candidate selected windows were retrieved based on the *Ensembl* cow genes database 104 using the *Ensembl* BioMart online tool (http://www.ensembl.org/biomart) [95]. We performed functional annotation of the *ZHp* detected gene sets in individual Sudanese zebu or the combined genesets using DAVID Bioinformatics online resources [96] for the identification of enriched gene ontology (GO) biological processes. We generated Venn diagrams based on the list of detected selective sweep regions and positively selected genes using the online tool at (https://molbiotools.com/listcompare.html).

Finally, following the overlap of *ZH*p and *F*_ST_ results, we further investigate the candidate selective sweep on chromosome 16 uniquely detected in five of the six Sudanese zebu breeds by performing the LD-based XP-EHH test using the rehh *ver* 2.0 R package [97]. Here, we contrasted the combined Group A Sudanese zebu breeds (except Fulani) against the Fulani, the other zebu breeds (Ethiopia Ogaden, Kenya Boran, and Gir), and the taurine breeds (African and European taurine). As this analysis required phased haplotype, we performed phasing of the entire chromosome 16 SNP data for all 150 cattle samples (excluding Group B Sudanese sequences) using the default parameter of Beagle ver 5.0 [98], except for *Ne*, which was set to 1000 to improve the accuracy of phase information according to Dutta et al. [22].

## Ethical approval

Not Applicable.

## Availability of data

The raw sequencing data for the Sudanese zebu cattle samples are available from the Sequence Read Archive (SRA) with the Bioproject accession number PRJNA858239. The accessions for the previously published datasets can be found in Supplementary Table 1

## Disclosures

The authors with this declare that there are no conflicts of interest, be it financial or otherwise.

## Author contributions

Author contributions: HM, OH, and AT designed the research; HM secured funding for the sampling and sequencing of the Sudanese zebu samples; HM, BS, HE, and TM collected the Sudanese samples; MVB contributed the data on Gir cattle from Brazil; KM secured funding for the analysis of the data; AT analysed the data; AT and OH interpreted the results; AT drafted the manuscript, AT, HM KM, and OH edited the manuscript; All authors revised and approved the manuscript's final version.

## Grants

This work is part of the project “agricultural growth, capacity building for scientific preservation of livestock breeds in Sudan”. The project was supported by a Korea-Africa Economic Cooperation Trust Fund grant through the African Development Bank (Grant No: KOAFEC-TF-2013).

This research was funded in part by the International Livestock Research Institute (ILRI) LiveGne program, supported by the CGIAR Research Program on Livestock (CRP livestock project) sponsored by the CGIAR funding contributors to the Trust Fund (http://www.cgiar.org/about-us/our-funders/). Also by the 10.13039/100000865Bill & Melinda Gates Foundation and with UK aid from the UK Foreign, Commonwealth, and Development Office (Grant Agreement OPP1127286) under the auspices of the Centre for Tropical Livestock Genetics and Health (CTLGH), established jointly by the University of Edinburgh, SRUC (Scotland's Rural College), and the International Livestock Research Institute. The findings and conclusions contained within are those of the authors and do not necessarily reflect the positions or policies of the Bill & Melinda Gates Foundation nor the UK Government.

## CRediT authorship contribution statement

**Abdulfatai Tijjani:** Conceptualization, Formal analysis, Data curation, Visualization, Writing – original draft, Writing – review & editing. **Bashir Salim:** Resources, Writing – review & editing. **Marcos Vinicius Barbosa da Silva:** Resources, Writing – review & editing. **Hamza A. Eltahir:** Resources, Writing – review & editing. **Taha H. Musa:** Resources, Writing – review & editing. **Karen Marshall:** Writing – review & editing, Funding acquisition, Supervision. **Olivier Hanotte:** Conceptualization, Writing – review & editing, Supervision, Resources. **Hassan H. Musa:** Conceptualization, Resources, Writing – original draft, Visualization, Funding acquisition, Writing – review & editing.

## Appendix A Supplementary data

Genomic signatures for drylands adaptation - Supplementary tables Image 1

Genomic signatures for drylands adaptation - Supplementary figures Image 2
