# Supplementary material for: Genomic signatures for drylands adaptation at gene-rich regions in African zebu cattle
Source: Genomics. 2022 Jul;114(4):None. doi: 10.1016/j.ygeno.2022.110423 (PMC9388378; doi:10.1016/j.ygeno.2022.110423)
Supplement: Supplementary file 2 — Genomic signatures for drylands adaptation - Supplementary figures [file mmc2.docx]

**Supplementary Figures**


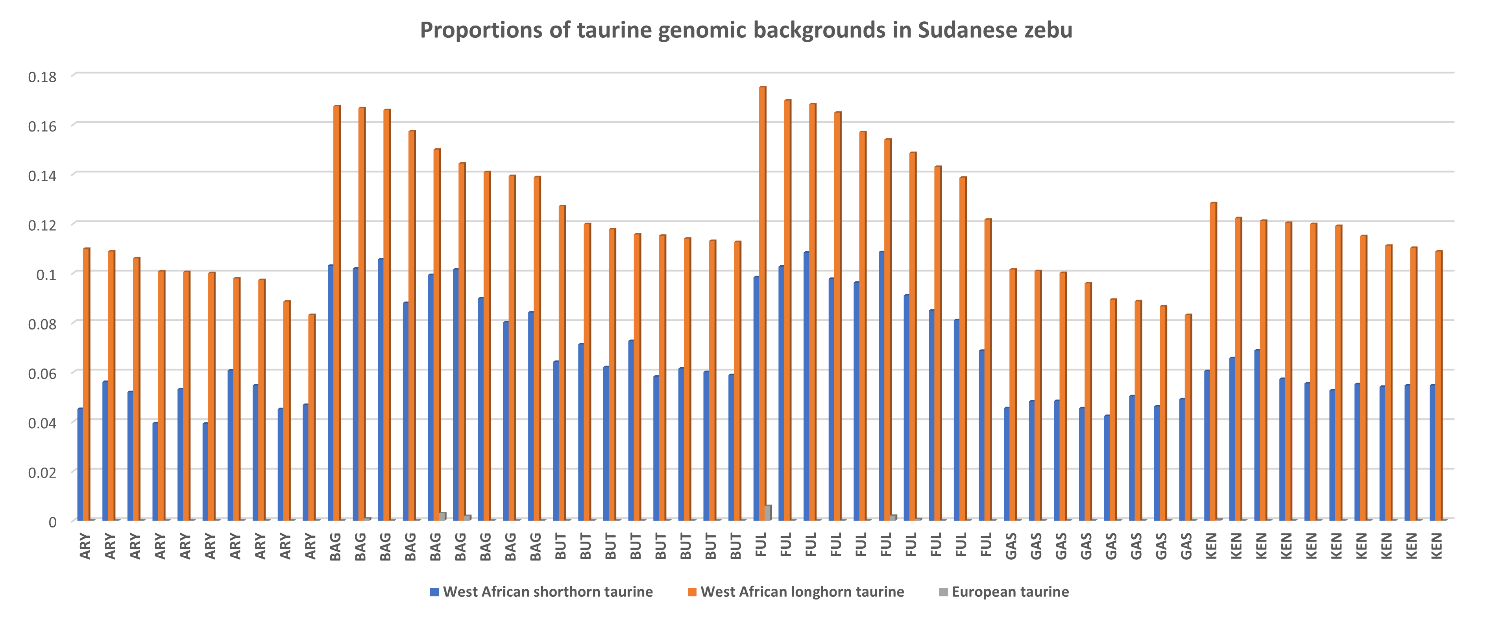


**Figure S1 –** Admixture proportions at K= 4, of taurine genomic backgrounds in individual Sudanese zebu. ARY – Aryashai, BAG – Baggara, BUT – Butana, FUL – Fulani, GAS – Gash and KEN – Kenana


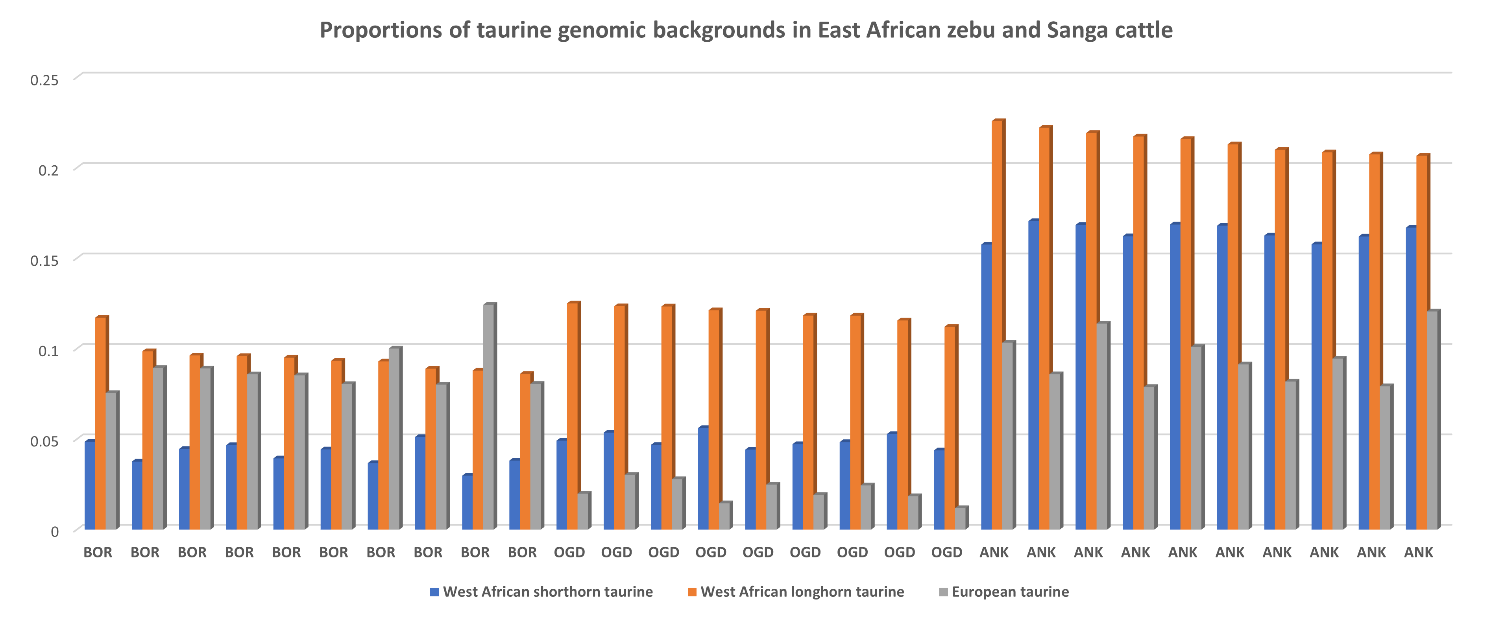


**Figure S2 –** Admixture proportions at K = 4, of taurine genomic backgrounds in East African zebu (BOR – Kenya Boran, OGD – Ogaden) and Sanga (ANK – Ankole) individuals.


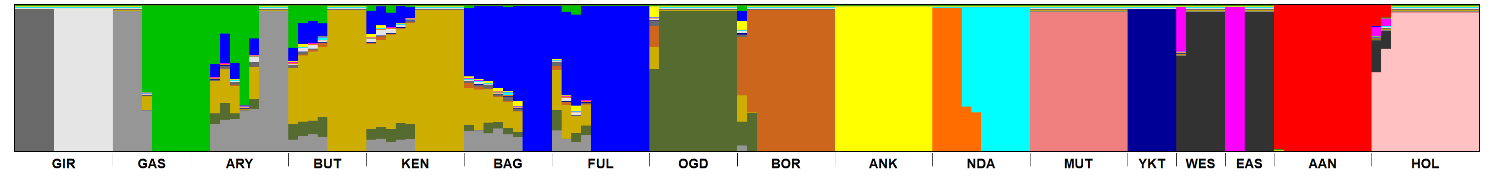


**Figure S3 -** Admixture analysis at K (17) equal to the number of breeds studied. The population structure was assessed using ADMIXTURE ver.1.3.0. The individual population is represented by a vertical bar and partitioned into coloured segments. GIR – Gir, GAS – Gash, ARY – Aryashai, BTN – Butana, KEN – Kenana, BGR – Baggara, FLN – Fulani, OGD – Ogaden, BOR – Kenya Boran, ANK – Ankole, NDA – N’Dama, MUT – Muturu, YKT – Yakutian, WES – Western Finncattle, EAS – Eastern Finncattle, AAN – Angus, and HOL – Holstein.


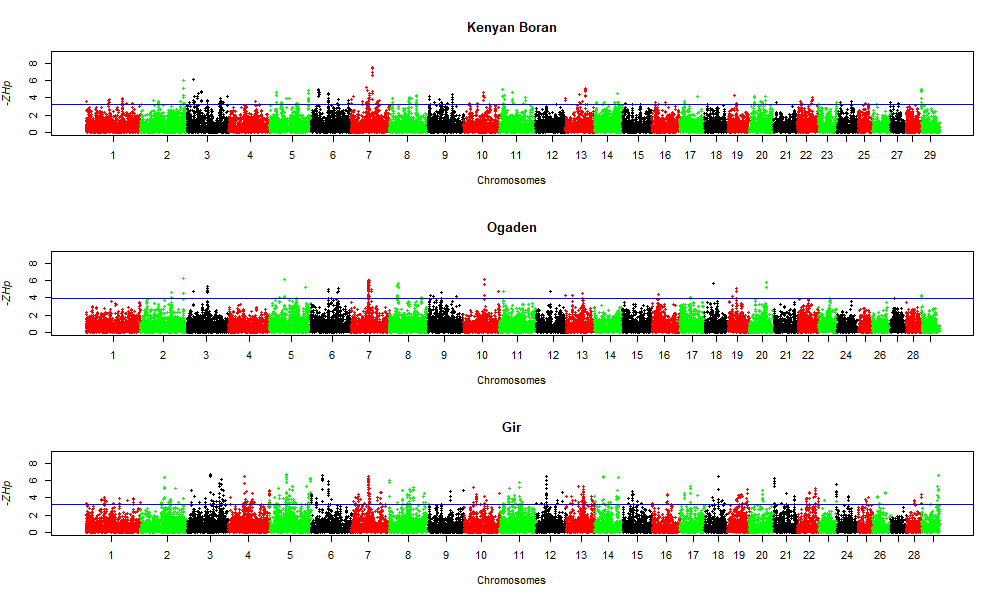


**Figure S4 -** The distributions of Z-transformed Hp scores across the bovine autosome in three zebu breeds (non-Sudanese); Kenya Boran (A), Ethiopian Ogaden (B) and Gir from Brazil (C). The blue line indicates the *ZH*p threshold value (lowest 0.5%) for selecting outlier windows (candidate regions under positive selection).


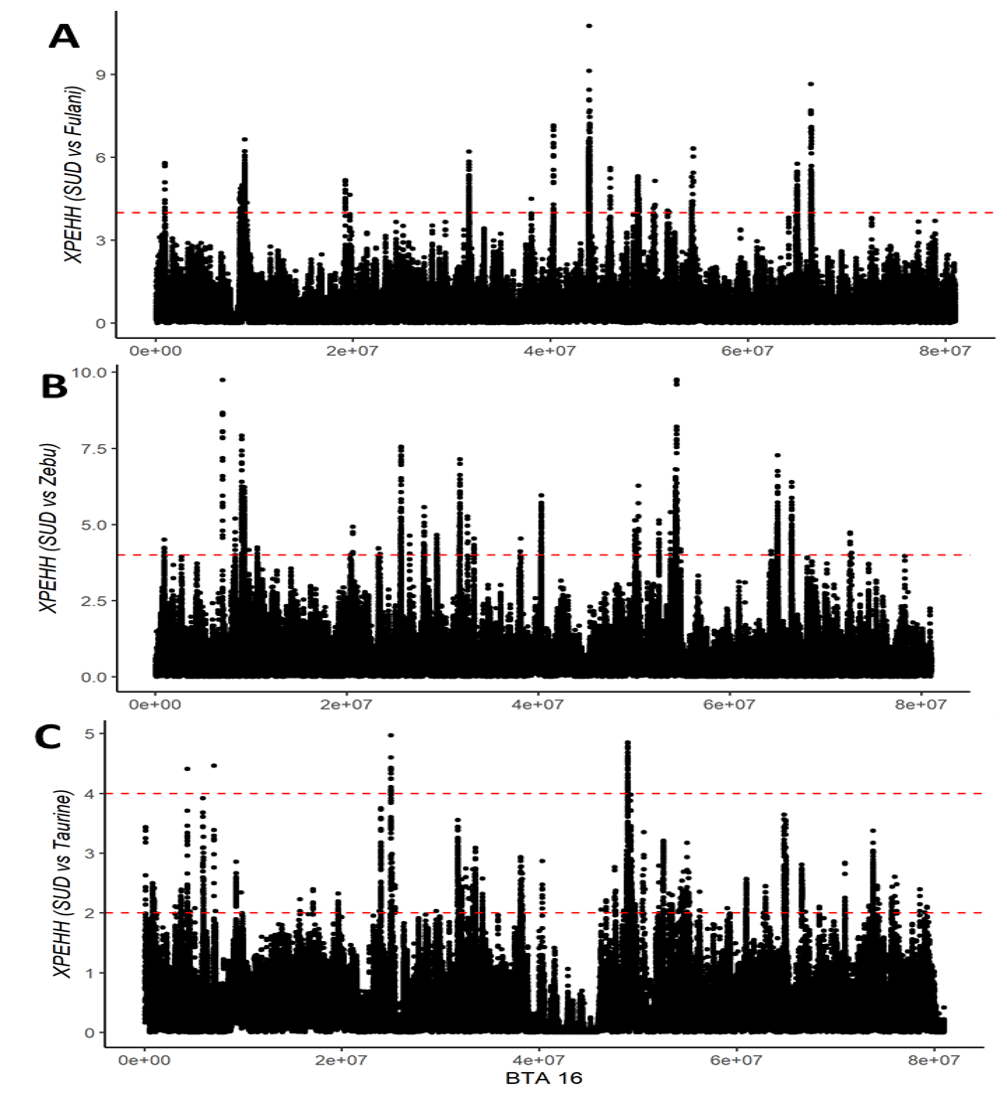


**Figure S5** – The distribution of XP-EHH scores along Bovine chromosome 16 following the comparison of the Sudanese zebu population with other zebu and taurine cattle populations. Sudanese Fulani (A), other zebu breeds (East African and Gir zebu) (B), and Taurine breeds (West African and Euroasia and West Europe breeds) (C). The red dash blue line indicates the significant XP-EHH threshold of >4 (*p*_value <0001) for selecting outlier SNPs (SNPs targeted by positive selection).


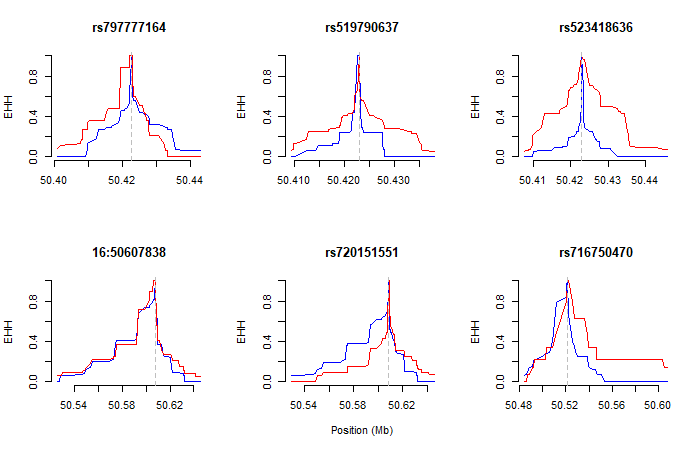


**Figure S6A** – The decay of extended haplotype homozygosity around six significant SNPs in the Sudanese zebu population.


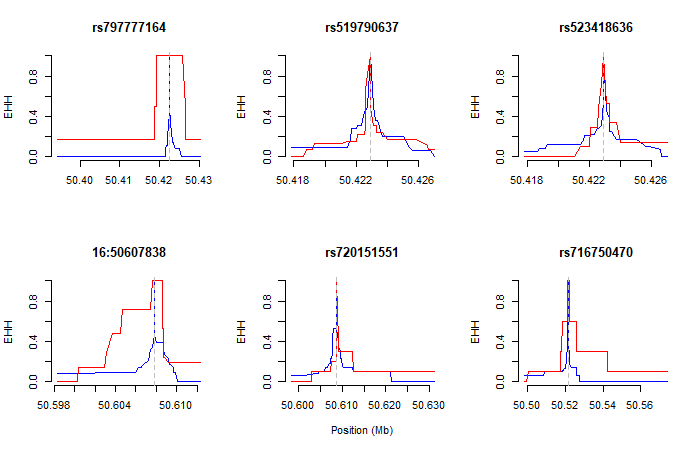


**Figure S6B** – The decay of extended haplotype homozygosity around six significant SNPs in Sudanese Fulani.


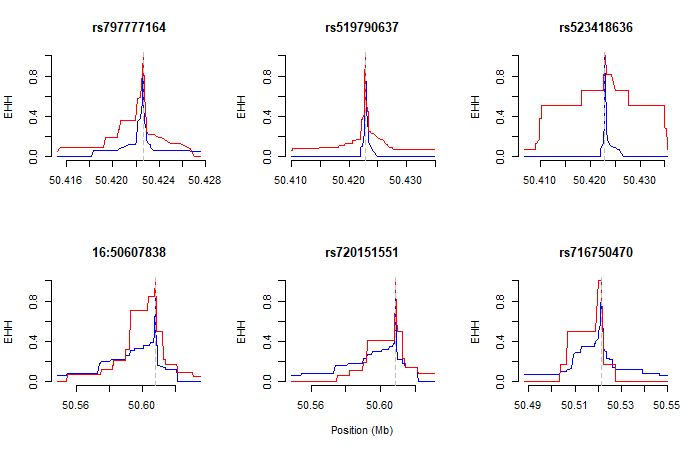


**Figure S6C** – The decay of extended haplotype homozygosity around six significant SNPs in other zebu population.
